# A Systematic Literature Review of the Epidemiology, Health-Related Quality of Life Impact, and Economic Burden of Immunoglobulin A Nephropathy

**DOI:** 10.36469/001c.26129

**Published:** 2021-09-01

**Authors:** Christina Soeun Kwon, Patrick Daniele, Anna Forsythe, Christopher Ngai

**Affiliations:** 1 Purple Squirrel Economics, New York, NY, USA; 2 Calliditas Therapeutics, New York, NY, USA

**Keywords:** epidemiology, end-stage renal disease, glomerular nephropathy, chronic kidney disease, renal biopsy, immunoglobulin a nephropathy

## Abstract

**Introduction:** This systematic literature review analyzed published evidence on IgA nephropathy (IgAN), focusing on US epidemiology, health-related quality of life (HRQoL), and economic burden of illness.

**Methods:** Using Preferred Reporting Items for Systematic Reviews and Meta-Analyses guidelines, Embase®, MEDLINE®, Cochrane, and Econlit (January 2010 to June 2020) were searched, along with relevant congresses (2017-2020).

**Results:** Of 123 epidemiologic studies selected for data extraction, 24 reported IgAN diagnosis rates ranging from 6.3% to 29.7% among adult and pediatric patients undergoing renal biopsy, with all reported US rates <15%. No US studies reported IgAN prevalence. A meta-analysis of US studies calculated an annual incidence of 1.29/100 000 people, translating to an annual US incidence of 4236 adults and children. Relative to Europe, the United States had more patients diagnosed with IgAN in later chronic kidney disease stages. US rates of transition to end-stage renal disease (ESRD) ranged from 12.5% to 23% during 3-3.9 years of observation, rising to 53% during 19 years of observation. Across 8 studies reporting HRQoL, pain and fatigue were the most reported symptoms, and patients consistently ranked kidney function and mortality as the most important treatment outcomes. Patients with glomerulopathy reported worse mental health than healthy controls or hemodialysis patients; proteinuria was significantly associated with poorer HRQoL and depression.

**Conclusion:** While economic evidence in IgAN remains sparse, management of ESRD is a major cost driver. IgAN is a rare disease where disease progression causes increasing patient burden, underscoring the need for therapies that prevent kidney function decline and HRQoL deterioration while reducing mortality.

## INTRODUCTION

Immunoglobulin A nephropathy (IgAN) is a rare, progressive autoimmune kidney disease that leads to chronic inflammation in the kidney.^1–3^ IgAN, a subtype of glomerulonephritis (GN), is induced by the accumulation of IgA-containing immune complexes in the kidney glomeruli that initiate a cascade of inflammatory events, eventually causing irreversible glomerulosclerosis that leads, in many patients, to end stage renal disease (ESRD), reduced quality of life (QoL), a need for dialysis or transplantation, and risk of premature death.^2,4^ The global incidence of IgAN among adults was estimated in one previous systematic literature review (SLR) to be ≥2.5/100 000/year.^5^

IgAN epidemiology, clinical presentation, and long-term outcomes vary across populations.^6,7^ IgAN is the most prevalent primary glomerular disease in East Asian and European populations, accounting for 30% to 45% of cases across Asia and Europe.^6,8^ Due to the lack of immediate signs and symptoms, many patients are not diagnosed until they present with evidence of chronic kidney disease (CKD), including hematuria, proteinuria, and severe hypertension.^2,3,9^ Kidney biopsy is required for definitive diagnosis of IgAN, leading to the potential for underdiagnosis or diagnostic delay in regions with limited screening, particularly for patients without severe symptoms.^2,3^

It has been reported that up to 50% of patients with IgAN progress to ESRD within 20 years of clinical presentation.^4,10–12^ Risk factors for progression to ESRD include persistent proteinuria, hypertension, and reduced glomerular filtration rate.^6,9^ Consequences of advanced CKD and kidney failure in IgAN include requirement for dialysis or transplantation, poor health-related quality of life (HRQoL), and increased mortality.^3,4,13–15^ Symptoms of CKD and adverse events due to dialysis have a substantial impact on HRQoL, daily living, and ability to work.^13,14^ Moreover, patients who undergo transplantation remain at risk of IgAN recurrence due to the underlying pathophysiology.^16^ All GN subtypes have the potential to recur post- transplantation, with the prevalence of GN recurrence between 3% and 15%.^17^ In addition, kidney transplant patients must deal with the associated life-long regimen of medications to reduce the risk of graft rejection, lifestyle changes, self-care, and medical appointments required to maintain the transplant.^18^

Current therapeutics, including supportive care and lifestyle modifications, address IgAN signs and symptoms without targeting underlying immune-mediated pathogenesis.^6,19^ Pharmacologic management of IgAN includes renin-angiotensin-aldosterone system (RAS) blockade with angiotensin-converting enzyme inhibitors (ACEi) and angiotensin receptor blockers (ARBs), with some use of aldosterone antagonists also reported.^19–21^ Late-stage clinical development is underway for a novel, targeted-release, orally administered glucocorticosteroid—budesonide—as the first IgAN-specific treatment targeting biochemical pathways implicated in IgAN pathophysiology.^22,23^

While the pathophysiology and clinical consequences of IgAN are well understood, the epidemiology, HRQoL, and economic burden of illness (BOI), as well as treatment patterns in the United States are less clearly described. Two previous SLRs focused on the global epidemiology of IgAN, covering published evidence from 1968 through 2017.^5,24^ However, there are no published SLRs examining the BOI or current treatment patterns in IgAN, nor are there published SLRs with a focus on the United States.

The objective of the current SLR was to gather and analyze published evidence on the epidemiology, burden, and current treatment patterns of IgAN. A secondary objective was to review published epidemiology evidence to estimate current US IgAN incidence.

## METHODS

### Data Review and Extraction

A two-part SLR (epidemiology and BOI) was conducted in accordance with the Preferred Reporting Items for Systematic Reviews and Meta-Analysis (PRISMA) guidelines.^25,26^ The methods followed the principles outlined in the Cochrane Handbook for Systematic Review of Interventions, the Centre for Reviews and Dissemination’s Guidance for Undertaking Reviews in Health Care, and the Methods for the Development of the National Institute for Health and Care Excellence’s Public Health Guidance.^26–28^ CSK was in charge of the final decision on study selection and quality control on data extraction. PD managed the meta-analysis. AF and CN managed the project.

### Data Sources

Key biomedical literature databases (Medical Literature Analysis and Retrieval System Online [MEDLINE®] and Excerpta Medica Database [Embase®]) and Cochrane were searched via the Ovid platform (January 2010 to June 2020). QoL and economic data are considered time sensitive. In addition, epidemiologic data are susceptible to change as diagnostic methods improve.^2^ Therefore, this time frame was used to avoid including outdated information.

MEDLINE® Epub Ahead of Print, In-Process & Other Non-Indexed Citations were searched to ensure that non-indexed citations were retrieved. The Econlit database was searched for economic studies. Bibliographies of relevant meta-analyses and SLRs were searched to retrieve any additional literature that may have been missed from the Ovid search. Conference abstracts from the European Renal Association – European Dialysis and Transplant Association, American Society of Nephrology, National Kidney Foundation, and International Symposium on IgA Nephropathy were searched from 2017 to July 2020 to identify abstracts not yet indexed in Ovid or published as full text articles at the time of search.

### Search Strategy and Inclusion/Exclusion Criteria

The detailed search strategy and results are presented in the Supplemental Material. The scope of the SLRs was defined by Population, Intervention, Comparators, Outcomes, and Study Design (PICOS) criteria (Table 1). The population in the epidemiology SLR included patients aged ≥18 years with IgAN, while the BOI SLR included patients of any age. Studies with mixed populations of adults and children were included in the epidemiology SLR if the majority of patients were adults. The main search terms for the population included “glomerulonephritis”, “immunoglobulin A nephropathy”, and “IGA”. Interventions included any treatment or no treatment; studies describing non-IgAN interventions and those with a population <20 patients were excluded. The epidemiology SLR included any publications that reported on incidence and prevalence, patient distribution across CKD stages, disease progression rate, treatment rate, and mortality in IgAN. Outcome measures in the BOI SLR included HRQoL, patient-reported outcomes (PROs), utilities, symptom burden, direct and indirect costs, and health-care resource utilization (HCRU). Various real-world evidence (RWE) study designs were included. Only English language studies were included. In the epidemiology SLR, only studies conducted in the United States, Canada, Western Europe, and Australia were selected for extraction. This decision was taken to limit heterogeneity in studies for 2 reasons: rates of screening for IgAN, epidemiology, and treatment patterns vary widely across regions, and a goal of the SLR was to identify evidence most relevant to the United States. In the BOI SLR, studies from all regions were selected for extraction to capture all available evidence in IgAN.

**Table 1.** PICOS Criteria for the (a) Epidemiology SLR and (b) BOI SLR

**a. Epidemiology SLR**		
**Category**	**Inclusion Criteria**	**Exclusion Criteria**
**Patient Population**	Adult patients (age ≥18) with IgAN regardless of comorbidities*	Non-human Pediatric patients (age <18) only Patients with other diseases
**Intervention**	Any treatment or management No treatment or management	Any non-IgAN treatment or management
**Outcomes Measures**	Incidence, prevalence, rate of IgAN Progression rate of IgAN through CKD stage and to ESRD Patient distribution across CKD stage Treatment rate Mortality	Studies that did not have ≥1 outcome in the inclusion criteria
**Study Design**	RWE studies including: Prospective observational studies Retrospective studies Registry analyses Database analyses Natural history studies and non-interventional studies SLRs and meta-analyses (for cross-checking only)	Reviews Editorials Notes/comments/letters Interventional studies Studies with <20 patients in the whole population Case reports/case series
**Restrictions**	English language Studies in the United States, Western Europe, or Australia Year limitation: 2010 to June 2020	Non-English language studies Studies outside of United States, Western Europe, or Australia
* Studies with mixed populations of adults and children were included Abbreviations: BOI, burden of illness; CKD, chronic kidney disease; ESRD, end-stage renal disease; IgAN, immunoglobulin A nephropathy; PICOS, Population, Intervention, Comparators, Outcomes, and Study Design; RWE, real-world evidence; SLR, systematic literature review.		
		
**b. BOI SLR**		
**Category**	**Inclusion Criteria**	**Exclusion Criteria**
**Patient Population**	Patients with IgAN regardless of comorbidities	Non-human Patients with other diseases
**Intervention**	Any treatment or management No treatment or management	Any non-IgAN treatment or management
**Outcomes Measures**	HRQoL outcomes PROs Utilities Symptom burden Costs (direct and indirect)/HCRU BOI Other QoL or economic burden	Studies that did not have ≥1 outcome in the inclusion criteria
**Study Design**	RWE studies including: Prospective observational studies Retrospective studies Registry analyses Database analyses Natural history studies and non-interventional studies SLRs and meta-analyses (for cross-checking only)	Reviews Editorials Notes/comments/letters Interventional studies Case reports/case series
**Restrictions**	English language Year limitation: 2010 to June 2020	Non-English language studies
Abbreviations: BOI, burden of illness; HCRU, health-care resource utilization; HRQoL, health-related quality of life; IgAN, immunoglobulin A nephropathy; PRO, patient-reported outcome; QoL, quality of life; RWE, real-world evidence; SLR, systematic literature review.		

### Meta-Analysis

A meta-analysis was performed on selected studies reporting IgAN among patients undergoing renal biopsy for any reason, to calculate US IgAN incidence. The meta-analysis followed methods published by the Cochrane Collaboration guidelines, the Agency for Healthcare Research and Quality, and the UK National Institute for Health and Care Excellence.^26–28^ Additional exclusion criteria applied to the selection of studies and data for meta-analysis were:

1. If more than one study used the same database, the study with the most recent data was selected.
2. If a study reported IgAN incidence rates during several time periods, data for the most recent time period were selected.
3. If a study reported IgAN incidence rates among multiple study populations (eg, adult-only or adult and pediatric), the rate among the largest study population was used.

Pooled proportions were estimated using a generalized linear mixed model meta-analysis framework with a logit transformation. Confidence intervals were estimated using the Clopper-Pearson or exact binomial method. Due to significant heterogeneity, the final estimate is based on random effects models. Analyses were conducted in R statistical software using the “meta” package with the “metaprop” function.

## RESULTS

### Epidemiology of IgAN

**Figure 1.**
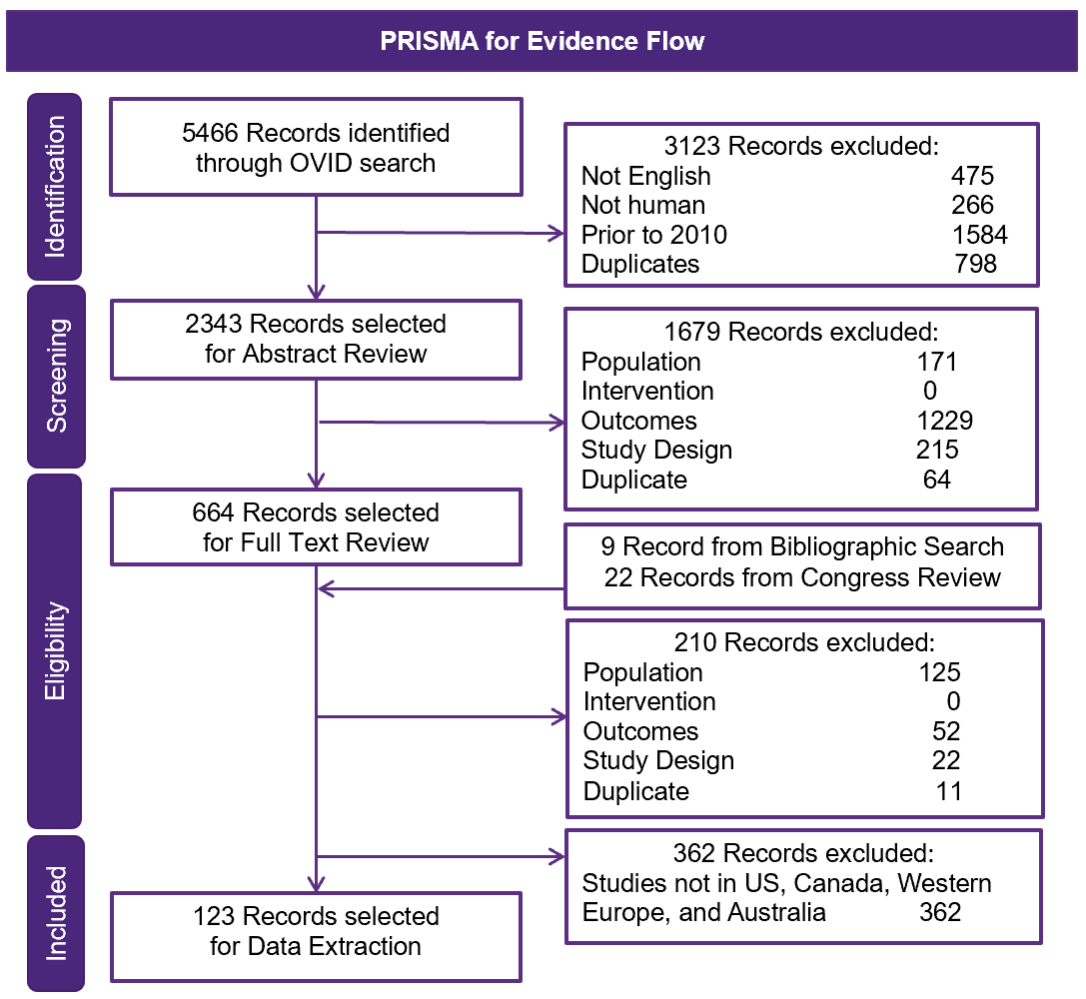
Study Selection for the Epidemiology SLR

**Figure 2.**
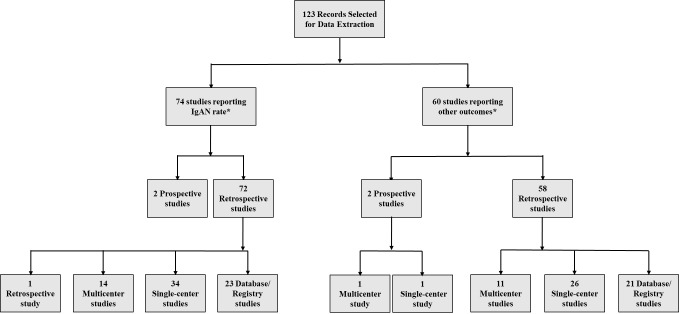
Study Design Distribution Among Included Epidemiologic Studies

Overall, 5466 publications were identified using the Ovid platform. Of these, 664 records were selected for full-text review, after screening by title and abstract. Following full-text review and the addition of studies from congress review and bibliographic search, a total of 123 records in the United States, Canada, Western Europe, and Australia were finally selected for data extraction (Figure 1). Seventy-four publications reported on diagnosis rates, incidence, or prevalence of IgAN (Figure 2). Most epidemiologic studies were conducted in the United States (n=16), followed by the United Kingdom (n=9) and Spain (n=8). Across 24 studies with a study population ranging from 83 to 33 391, the rate of IgAN diagnosis following renal biopsy was 6.3% to 29.7% among adults and children.^29,30^ Sixteen studies (n=200 to 21 374) reported an IgAN diagnosis rate of 9.2% to 34.6% among adults and children with GN or glomerular disease.

Eight studies reported incidence rates of IgAN per 100 000 persons/year, ranging from 0.7 (2000 to 2011) to 2.3 (2018) in the United States; rates in Italy, Canada, Germany, the Czech Republic, and Norway fell within this spectrum.^29,31–37^

**United States:** Of 16 total studies on IgAN rates in the United States, 6 reported IgAN rates among adults and children who underwent renal biopsy, ranging from 6.3% to 14.3% in study populations from 83 to 33 391 patients.^31,33,38–40^ Moreover, three US studies (n=710 to 21 374) reported IgAN rates of 9.4% to 19.7% among adults and children with GN or glomerular disease.^30,38,41^

One US study found an increasing trend in IgAN incidence, from 0.1/100 000 person-years in 2000 to 1.6/100 000 person-years in 2011.^31^ Three US-based studies reported IgAN rates among adult and pediatric patients with GN by race, finding higher rates in Asian Americans (2.75/100 000 person-years) than any other race (ranging from 0.1 in African Americans to 0.96 in Hispanic individuals).^31^ There were no US publications that reported on the prevalence of IgAN.

**Meta-analysis:** Five US studies reporting IgAN rates in patients with renal biopsy were selected for meta-analysis (as 2 of the 6 identified used the same database, the one with the longer study period was used) (Table 2). Based on an assumed US population of 328 200 000 according to US Census Bureau 2019 population estimates and a reported US renal biopsy rate of 15.8/100 000/year,^33,42^ US IgAN annual incidence was calculated as 1.29/100 000 persons, with an incident population of 4236 adults and children, in line with previously published rates (1.6 to 2.3/100 000/year).^31,33^

**Table 2.** US Studies Reporting IgAN Rates in Renal Biopsy

**Study Design and Reference**	**Study period**	**N**	**Diagnosis Rate of IgAN**	
Retrospective database (Kaiser Permanente, Northern California)^33^	2018	673	14.3%	
Retrospective single-center (University of North Carolina Chapel Hill Division of Nephropathology)^43^	1986 to 2015		6.6%	
Retrospective database (Kaiser Permanente Southern California Health System)^31^	2000 to 2011	4071	6.3%	
Retrospective, single-center (Saint Louis University Hospital)^39^	1999 to 2006	634	7.7%	
Retrospective, single-center (Rural center, Missouri)^40^	2013 to May 2019	118	8.4%	
**Meta-analysis of IgAN rates among patients with biopsy**		**38 887**	**8.17%**	
Abbreviations: IgAN, immunoglobulin A nephropathy; US, United States.				

**Disease progression and mortality:** 16 publications from the United States, Spain, France, Poland, Norway, Italy, and Sweden reported on CKD stage at the time of diagnosis or renal biopsy. Across studies, most patients were in CKD stage 1 (21.8% to 57.0%) or CKD stage 2 (20.0% to 44.4%) (Table 3). The United States had the highest proportion of patients in later CKD stages (29.1% in stages 4 and 5), suggesting delayed IgAN diagnosis.^44^ Spain had late-stage diagnosis rates of 16.6% to 23.3%, while other European rates were <9%.

**Table 3.** Distribution of IgAN Patients Across CKD Stages at Diagnosis/biopsy

**CKD Stage**	**Overall**	**US**		
1	21.8%-57.0%	22.7%, 23.5%		
2	20.0%-44.4%	20.0%, 23.9%		
3	6.3%-40.0%	24.3%, 40.0%		
4	1.3%-17.9%	17.9%		
5	0.9%-11.2%	11.2%		
Abbreviations: CKD, chronic kidney disease; IgAN, immunoglobulin A nephropathy; US, United States				

Thirty studies, including 6 from the United States, reported the rate of transition to ESRD in IgAN, which varied by endpoint definition, follow-up time, and patient characteristics. Reported US rates were 12.5% to 23% during 3 to 3.9 years of observation, rising to 53% over 19 years.^44–46^ Four studies discussed renal survival rates, including a US study reporting renal survival of 60% at 10 years and 50% at 18 years.^44^

Sixteen studies, including 5 from the United States, reported mortality rates in patients with IgAN. A 2018 study found a 10.1-year reduction in life expectancy relative to predicted age of death at time of kidney biopsy. Eighty-three percent of deaths in the study occurred after progression to ESRD.^44^

**Treatment patterns:** Twenty-four studies reported IgAN treatment patterns, which varied by country and patient characteristics. The most frequently used therapies were immunosuppressives, corticosteroids, and RAS blockers, including ACEi and ARBs. Six US-based studies found that 49.1% to 80% of IgAN patients were treated with immunosuppressive therapies, 47.2% to 61.5% with corticosteroids, and 80% to 90.5% with RAS blockers, including 67.7% with ACEi, and 37.9% with ARB.^30,44–49^

### Burden of Illness

**Figure 3.**
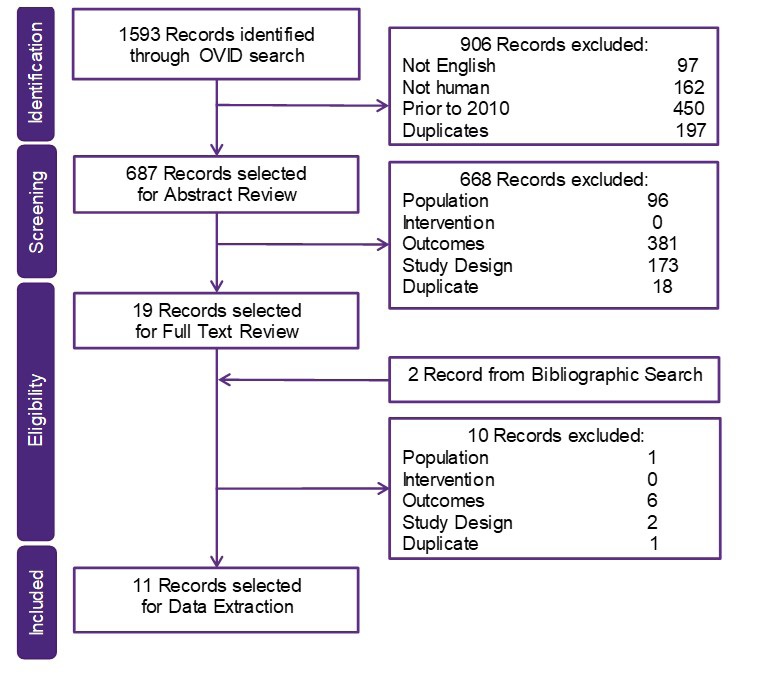
Study Selection for the BOI SLR

Overall, 1593 publications were identified in the BOI SLR, of which 19 were selected for full text review and 11 for data extraction (8 QoL, 3 economic) (Figure 3).

**Health-related quality of life:** Two of the extracted QoL studies were retrospective,^13,50^ one was a single-center prospective and observational study,^51^ four were patient surveys,^52–55^ and one was a validity study of a PRO assessment.^56^ These studies demonstrated that, even prior to progression to ESRD, patients with IgAN experience considerable symptom burden. The Short Form 36 Health Survey Questionnaire (SF-36) was used in two studies^51,54^ and other HRQoL instruments such as the Patient-Reported Outcomes Measurement Informative System (PROMIS),^13^ Pediatric Quality of Life Inventory (PedsQL),^55^ Hamilton Depression Rating Scale (HAMD),^54^ Beck Depression Inventory-II (BDI-II), Eysenck Personality Questionnaire (EPQ), Quality of Life Index (QLI), and Life Satisfaction Index (LSI),^51^ were used in one study each.

A retrospective analysis of 1336 social media postings of patients or caregivers from the United States and the United Kingdom (2017 to 2018) reported on the symptomatic burden of IgAN.^50^ Pain and fatigue were the most common symptoms associated with an impact on physical activity. Patients and caregivers reported emotional distress from lack of counseling or detailed information on IgAN. Patients were impacted by anxiety, depression, fear of progression to ESRD, the requirement for dialysis or transplantation, and the risk of IgAN recurrence post-transplant.^50^

Three non-interventional studies examined HRQoL and depression in glomerular diseases, including IgAN. An international longitudinal cohort study of children and adults with primary glomerular disease (126/478 children and 344/1115 adults had IgAN) found that edema was associated with poorer HRQoL as measured by PROMIS.^13^ A Brazilian study found that glomerulopathy patients (9/99 had IgAN) had lower mean SF-36 mental health scores than both healthy controls and matched hemodialysis patients (*P*<0.01).^54^ Patients with glomerulopathy were more depressed than healthy controls (*P*=0.002) as assessed by the Hamilton Depression Rating Scale, and proteinuria was significantly associated with poorer HRQoL and depression.^54^ A South African study of patients aged ≤18 years (mean age 14.4 years) with ESRD receiving dialysis (1/27 patients had IgAN) reported HRQoL scores measured by PedsQL that were significantly lower (*P*<0.05) than those of healthy children.^55^

Two studies examined the impact of lifestyle modification on HRQoL. A Japanese patient survey (n=81) identified no direct relationship between exercise intensity and decreasing levels of proteinuria, estimated glomerular filtration rate, or stress among outpatients with IgAN, while dietary improvements were associated with increased HRQoL (using an unspecified questionnaire).^53^ A prospective observational study from China demonstrated significantly improved depression levels and HRQoL among depressed adults with IgAN (n=108) who completed a personalized 6-month physical training program versus those receiving standard of care, using BDI-II and EPQ to measure depression levels and QLI, LSI, and SF-36.^51^

An interview-based study examined treatment outcomes in adults with glomerular disease and caregivers from Australia, Hong Kong, the United Kingdom, and the United States (18/134 had IgAN).^52^ Among patients, the highest-ranked outcomes (relative importance score from 0 to 1) were kidney function (0.40), mortality (0.29), need for dialysis or transplant (0.24), and life participation (0.18). Threats to future self and family, constraining day-to-day existence, and impaired agency and control over health were common concerns shared in patient and caregiver interviews.^52^

**Economic burden:** Two retrospective database studies reported HCRU and costs in IgAN. One study examined immunosuppressive medication costs in British Columbia, Canada, (756/2983 had IgAN) among adults with a diagnosis of GN on a native-kidney biopsy between January 2000 and December 2012.^57^ Among patients with IgAN, mean medication cost/patient/year did not significantly change from 2000 to 2013 (CAD158versusCAD221 in 2016 dollars; *P*=0.08). Prednisone was the most used immunosuppressive medication for IgAN, at 100% in 2000 and 89.3% in 2013.^57^ During this time, azathioprine usage decreased from 50.0% to 11.9%, while cyclophosphamide usage decreased from 25.0% to 4.8%.^57^

The approval of newer immunosuppressive medications with improved efficacy and less toxicity, such as mycophenolate mofetil, calcineurin inhibitors, and rituximab during this same timeframe may have contributed to reduced rates of utilization.^57^ Until recently, the benefits of immunosuppressive therapies for patients with IgAN were a source of debate.^58^

A database study from China discussed HCRU and costs among 11 569 hospitalized adults with IgAN between January 2010 and December 2015.^59^ Median inpatient costs were 8000 RMB (interquartile range [IQR]: 6000 to 12 000). Among all hospitalized patients with primary glomerular nephropathy, the proportion with IgAN decreased from 19.0% in 2010 to 10.6% in 2015. Routine admission was the most common type of hospital admission (86.5%; 95% CI 85.9, 87.2). Emergency and intensive care unit stays accounted for 8.0% (95% CI 7.5, 8.5) and 0.4% (95% CI 0.2, 0.5) of hospitalizations, respectively. Median length of stay was 10.0 days (IQR: 7.0 to 14.0).

The third study was a Japanese cost-effectiveness analysis that compared disease grade- and kidney function-based microsimulation methods to estimate cost-effectiveness in CKD, including IgAN.^60^ The study utilized previously reported health utility values and costs per year across CKD stages. Progression was associated with a decrease in health utility from 1.0 in stage 1 to 0.85 in stage 5.^60,61^ Dialysis was associated with a health utility of 0.72. Annual costs (2013 US dollars) increased from US$1600 in stage 1 to US$12 700 in stage 5.^62^ Dialysis incurred 50 times higher yearly costs relative to CKD stage 1 (US$1600 versus US$84 600).

## DISCUSSION

This SLR in IgAN examined epidemiology, treatment patterns, and HRQoL and economic burden across stages of disease progression, focusing on US evidence. A key challenge in establishing the incidence and prevalence of IgAN is the lack of a specific International Classification for Diseases 10^th^ revision (ICD-10) or 9th revision (ICD-9-CM) diagnostic code, meaning that large-scale database studies cannot directly identify IgAN cases. Moreover, IgAN is diagnosed via renal biopsy in some patients, but others do not have biopsy-confirmed diagnoses. Indeed, many patients with CKD and even ESRD have not undergone biopsy to establish a histopathologic diagnosis.^63^

The SLR found heterogeneous diagnosis rates across studies, from just over 6% of renal biopsies diagnosing IgAN to almost 30%. There are several factors known to impact IgAN diagnosis rates, including geography.^2^ Different countries provide varied access to primary care or guidelines on criteria for receiving renal biopsy.^2,64^ Many European and Asian countries have national health programs, which can increase referral rates to biopsy, in contrast to the cost and access barriers many patients must contend with in the United States. Besides geography, socioeconomic status, genetics, and race or ethnicity of patients could influence the diagnosis rates in the same country.^2^ For example, in the United Kingdom, where patients have access to the National Health Service, socioeconomic deprivation was associated with higher referral rate to renal biopsy and higher diagnosis rate of IgAN.^65^ Without free access to renal biopsies, detecting IgAN is more challenging.^2,65^ In the United States, IgAN rates among people who underwent renal biopsy were lower, with rates up to 15%, while the rate of IgAN diagnosis in Asian American patients was more than double that of any other race. A meta-analysis of US data confirmed the rarity of IgAN in the general population, calculating an IgAN annual incidence of 1.29/100 000 people and an incident population of 4236 adults and children. This finding is consistent with a previous SLR that covered published evidence from 1968 through 2017; the annual incidence rate of IgAN ranged from 0.54 to 2.1/100 000 people in three US regional studies (based on data from 1974 through 2003).^24^

The SLR did not identify any publications reporting on IgAN prevalence such that it was not feasible to calculate prevalence based on data available. One approach considered would have been to combine a publication of GN prevalence in the United States^66^ with IgAN estimates among the GN population from additional publications to estimate the proportion of GN cases that are specifically IgAN. However, there is considerable variability in the use of renal biopsy in diagnosing GN,^63^ so there would be substantial uncertainty associated with using a single published estimate of GN prevalence as the basis for an estimate.

Across studies, over half of IgAN patients progressed to ESRD, and in the United States, most deaths occurred after this progression. Of the countries studied, the United States had the highest proportion of IgAN detected in later CKD stages, indicating later diagnosis. Delayed diagnosis can lead to faster progression to ESRD, higher mortality rates, and higher economic burden.^2,64^ The proportion of patients diagnosed or biopsied at specific stages of CKD varied by country. This variation may be explained, at least in part, by differences in time to renal biopsy based on regional eligibility criteria for renal biopsy and/or access to primary care.^2^ Japan has broad screening programs during childhood, and IgAN is generally diagnosed at an early stage during routine checkups.^3,67^ A US retrospective study of the Nationwide Inpatient Sample from 2008 to 2012 found that more than half of the hospital admissions for percutaneous renal biopsy were covered by Medicare or Medicaid.^68^ In the United States, publicly insured patients face numerous barriers to health-care access that may substantially delay care, including diagnostic procedures.^69^ Since there is no consensus on renal biopsy indications or policies, renal biopsies are usually decided based on personal opinion and/or single-center policies.^70^ Therefore, higher incidence may be associated with heightened physician awareness in countries where IgAN is more common, and lead to earlier diagnoses. In addition, our analysis is also influenced by the inherent limitations of the evidence collected in terms of population size and characteristics as well as study design (ie, single center vs large registries or national databases).

RAS blockers were used by up to 90.5% of patients in US-based studies, consistent with Kidney Disease: Improving Global Outcomes (KDIGO) guideline recommendations.^6,19,71^ However, more than half of patients in US-based studies received immunosuppressive agents and systemic corticosteroids despite weak evidence supporting their use; often, these options are used in patients who, despite use of recommended therapies, show persistently high proteinuria levels.^6,19,72^ These findings reflect the lack of effective treatment options for IgAN. We found heterogeneity in treatment patterns by country despite similarities in clinical recommendations for various geographies.^3,67,73^ Though there is a paucity of evidence regarding specific factors impacting treatment selection and utilization, we suggest such variability may be due to the following factors: differences in treatment reimbursement or insurance across regions; variability in proportion of patients with high risk of progression due to timing of diagnosis, genetics, etc; mixed results of clinical trials by region or country; and the heterogeneity inherent to RWE, such as differences in included patients or study time period. Additional research investigating the influence of various factors on treatment patterns across geographies is warranted.

Limited evidence has been published on the HRQoL and economic burden of IgAN, underscoring the need for additional research in this rare disease. The considerable physical and mental health burden of IgAN increases with disease progression, particularly when dialysis becomes necessary. Nonetheless, patients experience symptoms and declines in HRQoL at all stages; HRQoL is impacted by pain, fatigue, anxiety, depression, and fear of disease progression. Moreover, proteinuria is significantly associated with depression and HRQoL declines. There is evidence that exercise may improve depression and HRQoL, and dietary changes have been associated with HRQoL benefits. There is a lack of data focusing on the economic consequences of IgAN, both globally and in the United States. Costs increase substantially as patients progress through CKD stages, with dialysis being a major driver of costs.^62^

### Limitations

This study has several limitations. As a rare disease, there is limited literature on IgAN epidemiology and BOI, with variations in the real-world study populations, methods, and patient characteristics. Variable rates of renal biopsy likely contribute to differences in diagnostic pathways, complicating the interpretation of literature from different countries. The meta-analysis conducted in this study relied on single-center studies and state registries, which may be less directly comparable than larger nationwide studies. In addition, prevalence of IgAN within the United States could not be reliably estimated.

In the BOI SLR, HRQoL data were often not presented for IgAN alone, but across glomerular disease populations and across all regions. The scant published HRQoL and economic evidence on IgAN in the United States and worldwide highlights the need for more research in this area. Nonetheless, the evidence compiled in this study shows that as IgAN progresses, burden increases substantially. The tendency of IgAN to be diagnosed at later CKD stages in the United States suggests that kidney damage may be significant before treatment can begin, implying the need for increased awareness and urine screening for proteinuria. Current therapies do not address the underlying pathophysiology of IgAN, and progressive disease requires extremely costly and burdensome treatments such as dialysis or transplantation. Agents that target the underlying disease have the potential to reduce the rate of progression to ESRD and could potentially decrease the burden of IgAN.

## CONCLUSION

This SLR found that IgAN, while rare, is associated with poor QoL and high economic burden. While the evidence base, particularly relating to US epidemiology and burden, is limited, the published literature demonstrates that, as patients progress through CKD stages, costs increase and HRQoL declines. Over half of patients with IgAN eventually progress to ESRD, negatively impacting QoL and increasing mortality.

The results of this analysis demonstrated an annual IgAN incidence in the US of 1.29/100 000 people. With no approved treatments targeting the underlying mechanisms of disease, patients face compounding symptoms, poor mental health, and a 10-year reduction in life expectancy, with death typically occurring after progression to ESRD. Patients consistently rank kidney function and life expectancy as the most important treatment outcomes, highlighting the need for a therapy to slow kidney function decline, preserve QoL, and reduce the risk of IgAN-related mortality. There are opportunities for additional research to expand the body of literature on IgAN, particularly in epidemiology, HRQoL and economic burden, and US-specific data.

## Supplementary Material

Online Supplementary Material
