# Supplementary material for: A Systematic Literature Review of the Epidemiology, Health-Related Quality of Life Impact, and Economic Burden of Immunoglobulin A Nephropathy
Source: J Health Econ Outcomes Res. 2021 Sep 1;8(2):36–45. doi: 10.36469/001c.26129 (PMC8410133; doi:10.36469/001c.26129)
Supplement: Online Supplementary Material [file jheor_2021_8_2_26129_70934.pdf]

Journal of Health Economics and Outcomes Research

Supplementary Online Material

Kwon CS, Daniele P, Forsythe A, Ngai C. A Systematic Literature Review of the Epidemiology, Health-Related Quality of Life Impact, and Economic Burden of Immunoglobulin A Nephropathy. *JHEOR*. 2021;8(2)36-45. doi:10.36469/jheor.2021.26129

SLR Methodology
Table S1: Search Strategy for Epidemiology
Table S2: Search Strategy for Burden of Illness

This supplementary material has been provided by the authors to give readers additional information about their work.

This is an open-access article distributed under the terms of the Creative Commons Attribution 4.0 International License (CCBY-4.0). View this license's legal deed at http://creativecommons.org/licenses/by/4.0 and legal code at http://creativecommons.org/licenses/by/4.0/legalcode for more information.

SLR Methodology: The methodologic approach followed three steps:

- Titles and abstracts of publications identified in Ovid searches were first independently assessed by two reviewers against pre-defined inclusion and exclusion criteria. Any discrepancies between the reviewers were reconciled by a third reviewer.
- Studies selected for inclusion during the title/abstract review were then assessed based on their full-text publication. Excluded publications were recorded with a justification as per the National Institute for Health and Care Excellence Decision Support Unit guidance.
- All studies selected for inclusion following full-text review underwent data extraction. Data from included studies were extracted into a predefined template, ensuring uniform extraction and consistency across studies.

Table S1: Search Strategy for Epidemiology

Search Conducted	26-Jun-2020			
Databases Searched	EBM Reviews - Cochrane Database of Systematic Reviews 2005 to June 17, 2020, EBM Reviews - ACP Journal Club 1991 to May 2020, EBM Reviews - Database of Abstracts of Reviews of Effects 1st Quarter 2016, EBM Reviews - Cochrane Clinical Answers June 2020, EBM Reviews - Cochrane Central Register of Controlled Trials May 2020, EBM Reviews - Cochrane Methodology Register 3rd Quarter 2012, EBM Reviews - Health Technology Assessment 4th Quarter 2016, EBM Reviews - NHS Economic Evaluation Database 1st Quarter 2016, Econlit 1886 to June 18, 2020, Embase 1974 to 2020 June 25, Ovid MEDLINE(R) and Epub Ahead of Print, In-Process & Other Non-Indexed Citations, Daily and Versions(R) 1946 to June 25, 2020			
			Term	Hits
Disease: IgA Nephropathy	Medline	1	Glomerulonephritis, IGA/	12 383
	Embase	2	immunoglobulin A nephropathy/	17 735
	All	3	((IgA or IgA-associated or IgA-induced or IgA-related or IgAassociated or IgAinduced or IgArelated or Immunoglobulin A or Berger or Berger's or Bergers) and (Nephropath$ or Nephritis or Glomerulonephritis or Glomerulonephritides)).ti,ab.	21 412
		4	(Bergers Disease$ or Berger's Disease$ or Berger Disease$).ti,ab.	468
		5	IgAN.ti,ab.	6327
Patients with IgA Nephropathy		6	or/1-5	24 841
Study Design-RWE Study		7	epidemiologic studies/	216 391
		8	exp case control studies/ or exp case control study/	216 391
		9	exp cohort studies/	2 744 329
		10	cross-sectional studies/	559 530
		11	clinical study/	158 845
		12	family study/	26 030
		13	longitudinal study/	275 734
		14	retrospective study/	1 754 147
		15	prospective study/	1 149 348
		16	cross-sectional study/	688 104
		17	cohort analysis/	853 062
		18	(case control or case-control).ti,ab.	295 390
		19	(cohort adj (study or studies or analys*)).ti,ab.	537 959
		20	((follow up or follow-up or observational or uncontrolled or non randomi#ed or nonrandomi#ed or non-randomi#ed or epidemiologic*) adj (study or studies)).ti,ab.	615 132
		21	((longitudinal or retrospective or prospective or cross sectional or cross-sectional) and (study or studies or review or analys* or cohort*)).ti,ab.	3 807 708
		22	((non-interventional or pragmatic) and (study or studies)).ti,ab.	37 165
		23	(registry or register or database or claims or single center or single-center or multicenter or multi-center or natural history or survey or record$ or real world or real-world).ti,ab	5 537 302
		24	follow-up/	1 551 654
		25	cohort*.ti,ab.	1 654 637
		26	24 and 25	223 769
		27	or/7-23, 26	11 005 365

Table S1: Search Strategy for Epidemiology

RWE Studies in Patients with IgA Nephropathy	28	6 and 27	6129
Irrelevant Study Design	29	(addresses or bibliography or biography or case report or comment or congresses or consensus development conference or duplicate publication or editorial or guideline or in vitro or interview or lectures or letter or monograph or news or "newspaper article" or practice guideline or "review" or "review literature" or "review of reported cases" or review, academic or review, multicase or review, tutorial or twin study).pt.	9 306 598
	30	(animals/ not (humans/ and animals/)) or (animal/ not (human/ and animal/))	5 794 096
	31	case report/ or case reports/	4 594 458
	32	or/29-31	18 696 563
	33	28 not 32	5466
Limits	34	limit 33 to English language	4991
	35	limit 34 to human	4725
	36	limit 35 to yr="2010 -Current"	3141
FINAL SELECTED: RWE Studies Reporting Epidemiology in Patients with IgAN	37	Deduplicate	2343

Table S2: Search Strategy for Burden of Illness

Search Conducted	25-Jun-2020			
Databases Searched	EBM Reviews - Cochrane Database of Systematic Reviews 2005 to June 17, 2020, EBM Reviews - ACP Journal Club 1991 to May 2020, EBM Reviews - Database of Abstracts of Reviews of Effects 1st Quarter 2016, EBM Reviews - Cochrane Clinical Answers June 2020, EBM Reviews - Cochrane Central Register of Controlled Trials May 2020, EBM Reviews - Cochrane Methodology Register 3rd Quarter 2012, EBM Reviews - Health Technology Assessment 4th Quarter 2016, EBM Reviews - NHS Economic Evaluation Database 1st Quarter 2016, Econlit 1886 to June 18, 2020, Embase 1974 to 2020 June 24, Ovid MEDLINE(R) and Epub Ahead of Print, In-Process & Other Non-Indexed Citations, Daily and Versions(R) 1946 to June 23, 2020			
		Term		Hits
Disease: IgA Nephropathy	Medline	1	Glomerulonephritis, IGA/	12 380
	Embase	2	immunoglobulin A nephropathy/	17 732
	All	3	((IgA or IgA-associated or IgA-induced or IgA-related or IgAassociated or IgAinduced or IgArelated or Immunoglobulin A or Berger or Berger's or Bergers) and (Nephropath$ or Nephritis or Glomerulonephritis or Glomerulonephritides)).ti,ab.	21 409
		4	(Bergers Disease$ or Berger's Disease$ or Berger Disease$).ti,ab.	468
		5	IgAN.ti,ab.	6325
Patients with IgA Nephropathy		6	or/1-5	24 838
QOL Outcomes	QOL	7	"quality of life"/	685 023
		8	"quality of life".de.	685 023
		9	(QOL* or HQL* or HQOL* or H QOL* or HRQL* or HRQOL* or HR QOL*).ti,ab.	184 462
		10	(quality adj4 life).ti,ab.	843 584
		11	(quality adj2 well?being).ti,ab.	963
	PRO	12	Patient Reported Outcome Measures/	26 195
		13	patient-reported outcome/	28 360
		14	(patient adj2 reported adj2 outcome$).ti,ab.	58 615
		15	(preference* adj4 (patient* or public or valu* or measur*)).ti,ab	66 987
		16	PRO.ti,ab.	502 479
	Utility	17	(utilit$ or disutilit$).ti,ab.	543 709
		18	(standard adj1 gamble*).ti,ab.	2147
		19	(("time trade" adj1 off*) or (time adj1 tradeoff*) or timetradeoff).ti,ab.	3819
		20	(willingness adj4 pay).ti,ab.	21 681
		21	(SG or TTO or WTP).ti,ab.	36 124
		22	((valu* or measur*) adj4 (health or outcome or outcomes or effect or effects or change* or state*)).ti,ab	1 321 280
		23	(euroqol$ or (euro adj1 qol$) or euro-qol$ or euroqual$ or (euro adj1 qual$) or euro-qual$ or eq5d$ or eq 5d$ or eq-5d$).mp,af,tw.	43 316
		24	(VAS or visual analogue scale$ or visual-analogue scale$).ti,ab.	199 562
		25	(Health Utilities Index or hui*).ti,ab.	11 379
		26	(multiattribute* adj1 (health or theor* or analys*)).ti,ab.	92
		27	((multi adj1 attribute*) and (attribute* adj1 theor*)).ti,ab.	4
		28	((multi adj1 attribute*) and (attribute* adj1 analys*)).ti,ab.	28
		29	((multi adj1 attribute*) and (attribute* adj1 health)).ti,ab.	90
		30	(Productivit$ or WPAI or work$ or employ$).ti,ab.	4 958 120
		31	sickness impact profile/	10 036

Table S2: Search Strategy for Burden of Illness

		32	(sickness impact profile or SIP).mp,af,tw.	19 960
	QoL measurements	33	(sf-36$ or sf36$ or sf 36$ or sf-12$ or sf12$ or sf 12$ or sf-6$ or sf6$ or sf 6$ or short form$ or shortform$ or RAND).mp,af,tw.	190 021
		34	(KDQOL-SF or KDQOLSF or KDQOL or KDQOL-36 or KDQOL36 or Kidney Disease Questionnaire or KDQ or NHP or "Nottingham Health Profile" or CHEP or CHOICE Health Experience Questionnaire or WHOQOL-BREF or WHOQOLBREF or WHOQOL).mp,af,tw.	20 217
ECON Outcomes	Cost	35	exp health care cost/	362 802
		36	"Costs and Cost Analysis"/	105 334
		37	exp fee/	70 479
		38	cost.de	58 132
		39	cost*.ti,ab.	1 653 101
		40	fee*.ti,ab.	1 339 712
		41	(price or pricing).ti,ab	206 753
		42	"drug use".de	114 345
		43	"drug utilization".de	41 082
	HCRU	44	(resource$ adj4 use$).ti,ab	87 107
		45	(resource* adj4 usage).ti,ab	2067
		46	(resource* adj4 utili*).ti,ab	49 565
	BOI	47	burden$.ti,ab.	583 166
		48	or/7-47	1 1170 425
QOL or Econ Studies in Patients with IgA Nephropathy		49	6 and 48	1891
Irrelevant Study Design		50	(addresses or bibliography or biography or case report or comment or congresses or consensus development conference or duplicate publication or editorial or guideline or in vitro or interview or lectures or letter or monograph or news or "newspaper article" or practice guideline or "review literature" or "review of reported cases" or review, academic or review, multicase or review, tutorial or twin study).pt.	4 104 235
		51	(animals/ not (humans/ and animals/)) or (animal/ not (human/ and animal/))	5 793 464
		52	case report/ or case reports/	4 593 648
		53	or/50-52	13 901 217
		54	49 not 53	1593
Limits		55	limit 54 to English language	1496
		56	limit 55 to human	1334
		57	limit 56 to yr="2010 -Current"	884
FINAL SELECTED: QOL or Econ Studies in Patients with IgAN		58	Deduplicate	687
